# [Retracted] Exosomes from LNCaP cells promote osteoblast activity through miR-375 transfer

**DOI:** 10.3892/ol.2025.15388

**Published:** 2025-11-17

**Authors:** Su-Liang Li, Na An, Bing Liu, Sheng-Yu Wang, Jian-Jun Wang, Yun Ye

Oncol Lett 17: 4463–4473, 2019; DOI: 10.3892/ol.2019.10110

Following the publication of the above paper, it was drawn to the Editor's attention by a concerned reader that the western blot data for the CD9 and GM130 experiments shown in Fig. 1C on p. 4466 were very similar to data that had already been published in a paper in the journal *Oncotarget*, which featured some of the same authors. The authors responded to the query concerning this figure, and were willing to repeat the experiment on account of the re-use of these data; however, upon performing an independent analysis of the data in this paper in the Editorial Office, it also came to light that the cellular images in Fig. 4A on p. 4470 contained apparent anomalies, namely the repeated patternings of small areas of cellular data, both within the same figure panels and comparing across the ‘miR-175 mimics’ and ‘Control’ data panels.

Owing to the apparently anomalous presentation of data in Fig. 4, the Editor has decided that this paper should be retracted from the Journal. The authors were asked for an explanation to account for these concerns, but the Editorial Office did not receive a satisfactory reply. The Editor apologizes to the readership for any inconvenience caused.

